# Hydrogen Sorption Behavior of Cast Ag-Mg Alloys

**DOI:** 10.3390/ma15010270

**Published:** 2021-12-30

**Authors:** Adam Dębski, Sylwia Terlicka, Anna Sypien, Władysław Gąsior, Magda Pęska, Marek Polański

**Affiliations:** 1 Institute of Metallurgy and Materials Science, Polish Academy of Sciences, 25 Reymonta Street, 30-059 Kraków, Poland; a.debski@imim.pl (A.D.); s.terlicka@imim.pl (S.T.); a.sypien@imim.pl (A.S.); w.gasior@imim.pl (W.G.); 2 Department of Functional Materials and Hydrogen Technology, Military University of Technology, 2 Kaliskiego Street, 00-908 Warsaw, Poland; magda.peska@wat.edu.pl

**Keywords:** Ag-Mg alloys, hydrogen storage, DSC measurements, X-ray diffraction, intermetallics

## Abstract

In this paper, the hydrogen sorption properties of casted Ag-Mg alloys were investigated. The obtained alloys were structurally analyzed by X-ray diffraction (XRD) and observed by scanning electron microscopy (SEM). The study was carried out for four alloys from the two-phase region (Mg) + γ′ (AgMg_4_) with nominal concentrations of 5 wt. %, 10 wt. %, 15 wt. %, and 20 wt. % Ag, four alloys with nominal compositions equivalent to intermetallic phases: AgMg_4_, AgMg_3_, AgMg, and Ag_3_Mg, one alloy from the two-phase region AgMg + Ag_3_Mg (Ag60Mg40), and one alloy from the two-phase region AgMg + AgMg_3_ (Ag40Mg60). The hydrogenation process was performed using a Sievert-type sorption analyzer. The hydride decomposition temperature and kinetic properties of the synthesized hydrides were investigated by differential scanning calorimetry (DSC) coupled with thermogravimetric analysis (TGA). Samples with high magnesium content were found to readily absorb significant amounts of hydrogen, while hydrogen absorption was not observed for samples with silver concentrations higher than 50 at. % (AgMg intermetallic phase).

## 1. Introduction

Over the past few years, intensive studies have been carried out to promote the development of green technologies, such as hydrogen energy, in the framework of applications for the automotive sector and green energy sources. Hydrogen belongs to a group of flammable and explosive gases that require special precautions for safe storage. Hydrogen can be stored in compressed or liquid form, alternatively adsorbed on the surface of materials, or chemically bound in the form of hydrides. Hydrogen is widely stored in a compressed form but (although apparently unjustified) this is considered to be dangerous by society and uneconomical due to high compression costs with a relatively low volumetric capacity of approximately 40 kg/m^3^. Hydrogen storage in liquid form is even less cost-effective than storage in compressed form and requires the use of special cryogenic tanks at a temperature of 20 K. The final method of hydrogen storage is in the form of metal hydrides or intermetallic phases, which can contain ~80–150 kg of hydrogen per 1 m^3^ [1,2,3,4]. Therefore, this method is the most advantageous form of hydrogen storage, both in terms of safety (if properly used) and volumetric capacity.

Many metals or intermetallic phases easily absorb and desorb hydrogen, but due to their low gravimetric capacity, they are not currently considered for future mobile applications [5]. Magnesium-based hydrides are one of the most promising materials for hydrogen storage due to the high gravimetric capacity of hydrogen (up to 7.6 wt. % for MgH_2_) and relatively low cost [6,7,8,9]. However, because of the need to use high temperatures and the slow kinetics of the hydrogen absorption/desorption reaction, the use of Mg for hydrogen storage precludes its practical application. Therefore, intensive research is currently being conducted globally to improve the kinetics of hydrogen absorption/desorption in magnesium alloys by modification with alloying additives or catalysts [9,10,11,12,13]. In 2013, Si et al. [14] presented the first studies on hydrogen sorption for the Ag-Mg system. They investigated the AgMg_3_ intermetallic compound as potential material for hydrogen storage. The AgMg_3_ sample was prepared by reactive sintering of compressed pellets of Ag and MgH_2_ powders. It was demonstrated that the hydrogenation/dehydrogenation process for the AgMg_3_ intermetallic phase is reversible. It was observed that the thermodynamics of this process differ from that of pure magnesium, which results in different equilibrium pressures. Later, Urretavizcaya et al. [15] presented the results of hydrogen absorption and desorption in Mg and Mg-Ag compounds prepared by mechanical milling. They observed that the addition of silver to Mg slightly improves the hydrogen absorption and desorption kinetics, while further destabilization reactions were observed. Most recently, Pęska et al. [16] showed the relationships for mechanically synthesized magnesium-silver alloys after both milling and annealing. It was shown that all compositions with silver concentrations lower than that for AgMg readily react with hydrogen, although the mechanism is different for different compositions and in agreement with that reported previously by Si et al. and Urretavizcaya et al. [14,15]. No samples with concentrations higher than 50 at. % Ag were investigated. Additionally, it is worth noting that mechanical synthesis is a great tool for material synthesis, which allows for the manufacturing of very pure and difficult-to-cast compositions however, in real-life applications, casting is much cheaper and applicable on a large scale. Melting and casting is a technique that offers higher cost efficiency than mechanical synthesis methods but very often results in segregated samples.

As there is a lack of information in the literature for the interaction of hydrogen with Ag-Mg alloys and intermetallic phases prepared by classical metal melting, this study aims to provide such information.

## 2. Materials and Methods

**Figure 1:**
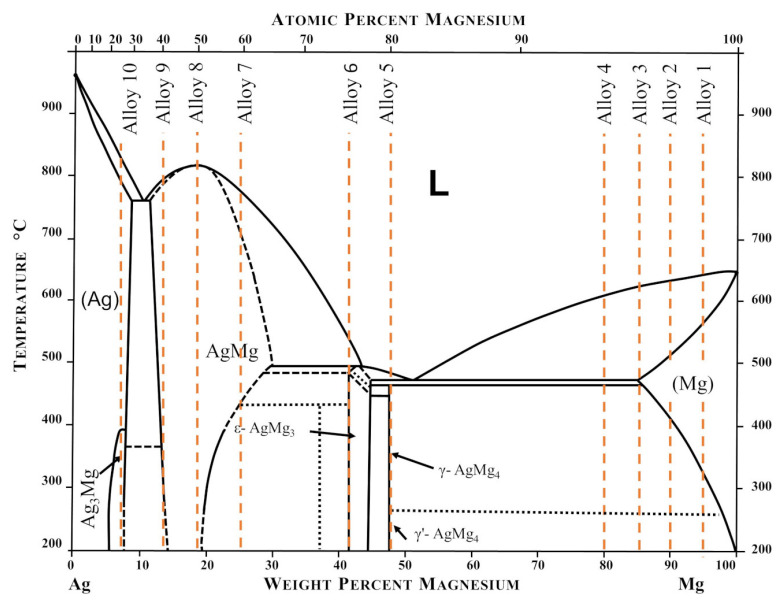
Representation of the chemical composition of the fabricated Mg-Ag samples on the Ag-Mg phase diagram. Diagram based on [16].

The alloys were prepared by conventional melting of the appropriate amounts of metals using tungsten crucibles (Spinex Spinkiewicz SJ, Warsaw, Poland) in a resistance furnace placed in a glove box filled with a protective high purity argon atmosphere (H_2_O < 0.5 ppm, O_2_ < 0.1 ppm, N_2_ < 1 ppm). The purity of the used materials is presented in Table 1. The liquid alloys were cast into steel ingot molds. Cast alloys with nominal concentrations of 5 wt. %, 10 wt. %, 15 wt. %, and 20 wt. % Ag were annealed at 699 K for 1 week. For the case of intermetallic phases and alloys in the two-phase region, prepared alloys were subjected to annealing for 744 h at 699 K for homogenization. The furnace was set to a temperature of 699 K. The temperature of the samples themselves was approximately 35–40 degrees lower than this temperature. Ten Ag-Mg samples were prepared, four with silver concentrations equal to 5 wt. %, 10 wt. %, 15 wt. %, and 20 wt. % Ag, four intermetallic phases such as γ′ (AgMg_4_), ε (AgMg_3_), AgMg, and Ag_3_Mg, and two alloys Ag40Mg60 and Ag60Mg40 from two-phase regions: AgMg + Ag_3_Mg and AgMg + AgMg_3_. To increase the readability of the description for the reader, and since it was discovered that not all of the samples obtained showed the expected phase composition, the samples in this work were named simply as Alloy 1–Alloy 10. The expected chemical composition of the alloys is shown in Figure 1.

After completing the homogenization process, all samples were prepared in the form of filings using a hand file. XRD phase analysis, both before and after hydrogenation, was performed for each sample. For these studies, an Ultima IV X-ray diffractometer (Rigaku, Tokyo, Japan, Co Kα radiation, 1.79026 Å) was used with operating parameters of 40 mA, 40 kV, and a scanning speed of 1 deg/min. For some of the samples, an X’Pert PRO diffractometer (PANalytical, Malvern, UK) was used with CuKα radiation. A graphite monochromator was used to reduce the Kβ fraction of the radiation. The morphologies of the cast Ag-Mg samples after filing were examined by using a Quanta 3D FEG scanning electron microscope (SEM) (FEI, Hillsboro, OR, USA) Microstructure and chemical composition analyses were carried out at an accelerating voltage of 15 kV and a current intensity of 11.3 nA. The hydrogenation process was carried out using a Sievert-type sorption analyzer (IMI-HTP, Hiden Isochema, Warrington, UK). Differential scanning calorimetry (DSC) analysis was conducted simultaneously with thermogravimetric analysis (TGA) for samples after heating under hydrogen pressure using a Setaram Sensys Evo 3D analyzer (Lyon, France) under a flow of ultrahigh purity helium (BIP, Air Products, Allentown, PA, USA). The device was set up in the vertical position, and each sample was loaded into an alumina crucible without air exposure. Before the start of heating, each sample was flushed with high purity helium for 2 h. The apparatus was calibrated for heating rate and energy with the use of high purity In, Sn, Pb, Zn, and Al standards. Additionally, before the experiment, a full-scale calibration of the thermobalance was performed using an E1 class mass standard (Radwag, Radom, Poland) with a mass of 200 mg. Each sample was heated to 450 °C with a 5-°C/min heating rate to observe decomposition.

## 3. Results and Discussion

### 3.1. Morphology and Phase Composition of Ag-Mg Alloys before Hydrogenation

**Figure 2:**
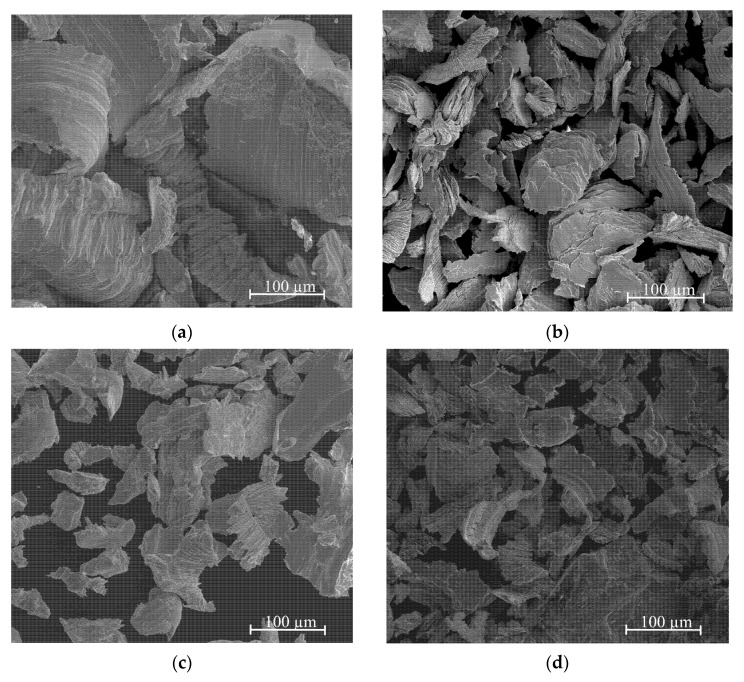

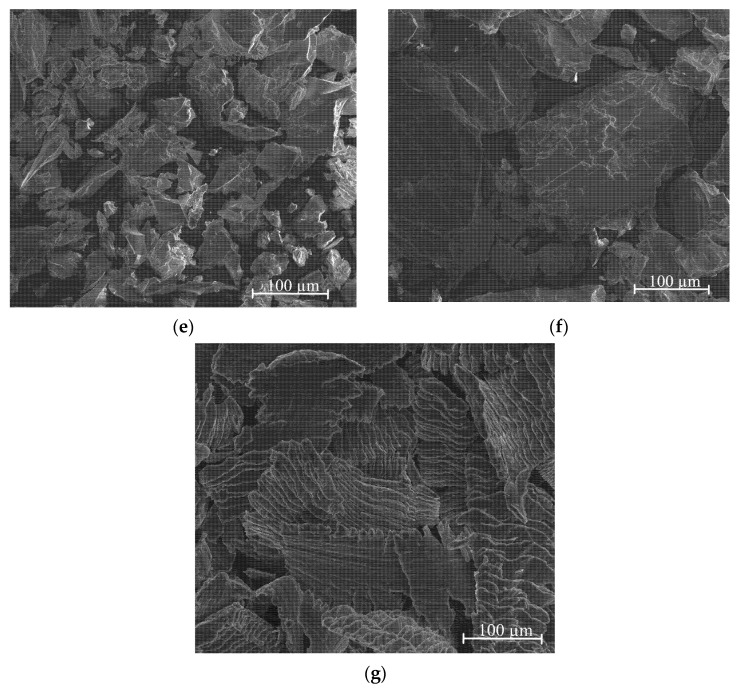
SEM microstructures of various Ag-Mg alloys: (**a**) Alloy 10, (**b**) Alloy 9, (**c**) Alloy 8, (**d**) Alloy 7, (**e**) Alloy 6, (**f**) Alloy 5, and (**g**) Alloy 2.

The obtained Ag–Mg alloys were characterized using scanning electron microscopy (SEM), and X-ray diffraction (XRD) patterns were collected to analyze the phase content. The morphology of the particles in the prepared samples of alloys is shown in Figure 2a–g. Despite using the same technique and operator, the form of prepared filings depends slightly on the alloy type. This should be treated as a semiquantitative observation but definitely cannot be neglected. For example, for Alloy 10 (Ag_3_Mg), the filings are wide, thin, and long, and for Alloy 9 (Ag60Mg40—two-phase region), most of the filings are much smaller. For the case of the Alloy 8 AgMg phase with a higher concentration than that mentioned above, the filings are comparable with those for Alloy 10 (Ag_3_Mg), and those for Alloy 7 (Ag40Mg60 two-phase alloy) are comparable to those for Alloy 9 (Ag60Mg40). The general conclusion is that filings produced from alloys belonging to the two-phase region are larger than those obtained for the intermetallic phases. This probably means that alloys from the two-phase region have slightly higher plasticity. Brittle cleavages were observed for the “e” and “f” samples, which is slightly unintuitive since one would expect high brittleness for AgMg and Ag_3_Mg samples.

Nevertheless, the main dimensions of the particles after the filling process range between tens and hundreds of microns. From our experience, this size range should be sufficient to promote easy hydrogenation, so no further processing (such as ball milling) was performed to avoid introducing more variables to the process (stress, strain, and a further increase in the specific surface area) and possible contamination.

**Figure 3:**
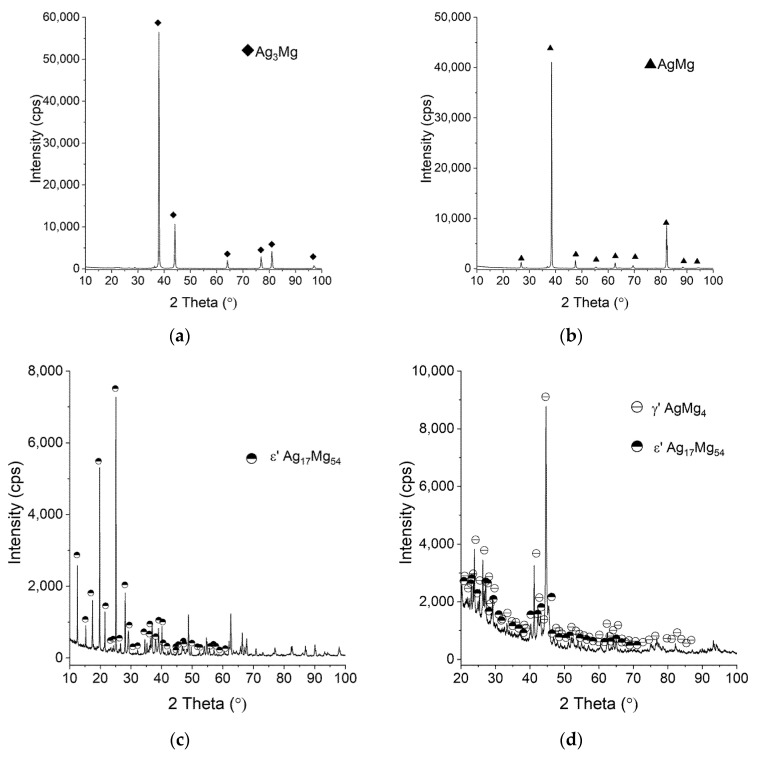
XRD patterns for Ag-Mg alloys: (**a**) Alloy 10 (**b**) Alloy 8 (**c**) Alloy 6, and (**d**) Alloy 5.

The XRD patterns for exemplary alloys with a nominal composition correspond-ing to Ag-Mg intermetallic phases (Ag_3_Mg, AgMg, ε-AgMg_3_, and γ′-AgMg_4_) are presented in Figure 3a–d. XRD studies confirmed the presence of the expected inter-metallic phases in the majority of the obtained samples. The major difference observed is the presence of an ε’ phase instead of the expected ε phase (Alloy 6). This phase has been found previously in cast alloys, as described by Arakcheeva et al. [17]. Later, Pęska et al. [16] observed mechanically alloyed and annealed samples and found that they readily react with hydrogen at elevated temperatures.

### 3.2. Hydrogen Absorption Studies of Analyzed Ag-Mg Alloys

**Figure 4:**
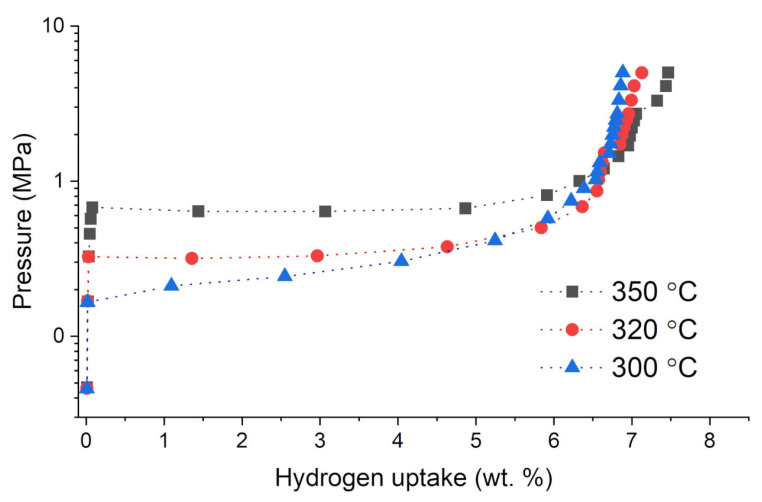
Hydrogen absorption isotherms for the Alloy 2 (10AgMg) sample as a representative for a group of alloys (5 wt. %, 10 wt. %, 15 wt. %, and 20 wt. % Ag).

**Figure 5:**
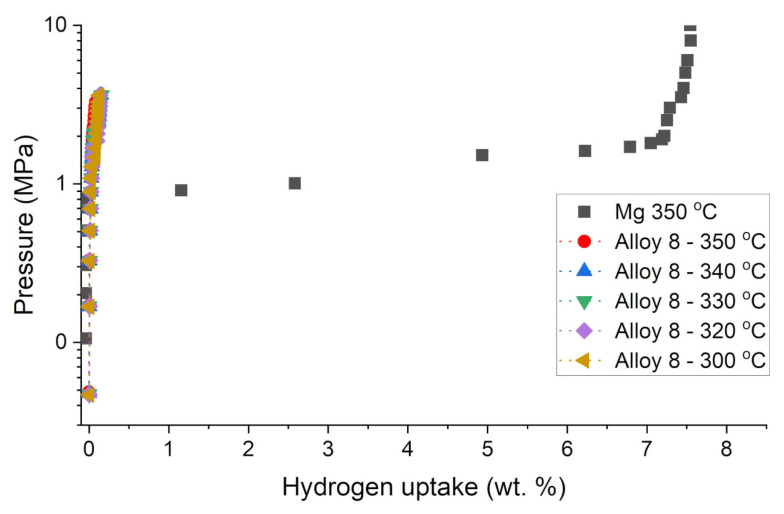
Hydrogen absorption isotherms for the Alloy 8 (AgMg) compared to isotherm of pure magne-sium sample at 350 °C.

Hydrogen absorption and desorption studies were performed using Sievert’s apparatus. All hydrogen sorption measurements for the as-prepared samples were performed in the same manner. The measurement was started by loading the powdered sample into the reactor and outgassing the sample to obtain a pressure of 1 × 10^−5^ mbar at the vacuum gauge. It must be stated that the vacuum gauge was placed close to the turbomolecular pump, while the sample was placed relatively far from the gauge and connected with relatively small diameter tubes. For that reason, the pressure in the reactor should be considered to be at least an order of magnitude higher than that on the gauge, with a high probability reaching much lower values than 1 × 10^−4^ mbar. The volume of the sample was measured by a pycnometry procedure with the use of high purity helium. Then, hydrogen was dosed into the reactor until the final pressure reached ~80 bar, and after that, the sample was heated to 400 °C at a rate of 10 K/min in a hydrogen atmosphere. Additionally, outgassing of the sample at 400 °C for 2 h was performed before each isotherm. This process was called activation and enabled one to obtain stable and active samples. After activation, isothermal measurements were performed for the chosen samples. The selected hydrogen sorption isotherms for the investigated alloys from the Ag-Mg system are shown in Figure 4 and Figure 5. Alloy 2 containing 10 wt. % Ag was chosen as an example for the group of alloys from the two-phase region (Mg) + γ′ (AgMg_4_). By analyzing Figure 1, it can be observed that the alloy contains 10 wt. % Ag, which enables maximum hydrogen absorption at 350 °C with a quantity very similar to that of pure Mg. Isotherms were performed at several temperatures below 300 °C. However, for the case of 300 °C, it became evident that the measurement points are kinetically influenced since the sample did not reach the equilibrium pressure at the given (reasonable) single point saturation times (90 min). Therefore, these results are not shown. Furthermore, the results (two-step plateau) confirm that hydrogen sorption proceeds in several steps, in which it is very likely that magnesium reacts with hydrogen to form magnesium hydride, and the ε or AgMg_4_ phases react with hydrogen to form stable AgMg and MgH_2_ phases, as mentioned previously [15,16]. As a result of these reactions, the AgMg phase formed in the system does not react with hydrogen and blocks the absorption and desorption processes.

Examples of hydrogen absorption isotherms for the Alloy 8 (AgMg phase) sample are shown in Figure 5. When the amount of absorbed hydrogen is close to zero, the values observed (not exceeding 0.15%) are probably influenced more by the cumulative error of the method rather than true absorption. Alternatively, these low values might be related to the presence of an insignificant amount of residual magnesium or hydrogen absorbing phase. The thermodynamic discussion and reaction mechanisms for Mg-Ag phases with hydrogen have already been shown in the literature [14,15,18,19], and for that reason, they will no longer be explored in this paper. We discuss only the kinetics of the absorption and the properties of the manufactured material.

**Figure 6:**
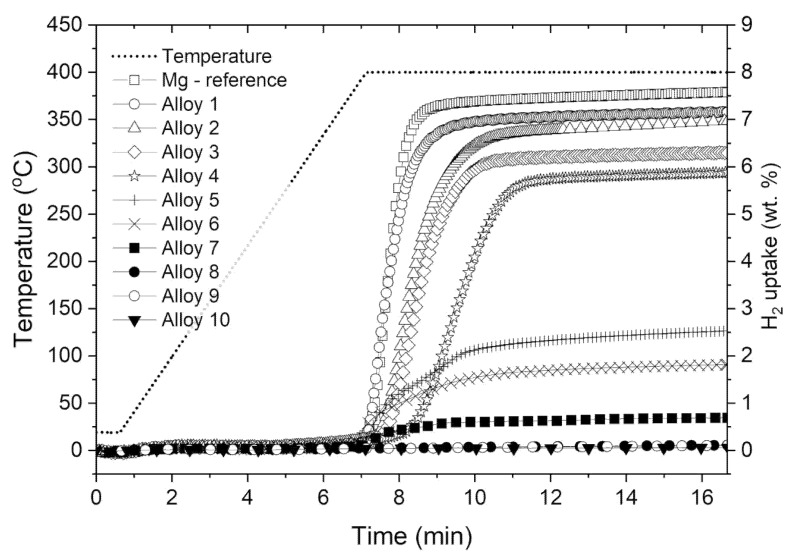
Comparison of the hydrogen absorption kinetics for the tested samples.

A comparison of the absorption study for Ag-Mg alloys is presented in Figure 6, where the kinetics of hydrogen absorption in Ag-Mg alloys are presented. The experiment was performed by first pressurizing samples to approximately 66 bar (hydrogen dosing). Then, the samples were heated to 400 °C at a rate of 1 °C/min in a hydrogen atmosphere. The final pressure was approximately 72 bar. From the analysis of Figure 6, the addition of silver to magnesium reduces the sorption capacity of the samples and has no significant effect on the hydrogen sorption kinetics however, samples from the hypoeutectic region (up to 20% Ag) show a rather negative correlation. The visible differences in kinetics may be due to, inter alia, differences in the particle size used in the experiments (different mechanical properties and different filing efficiencies, as already shown in Figure 2. It can be observed that all prepared samples show similar kinetics, which is most likely caused by the reaction of AgMg_4_ and/or ε phases and formation of AgMg and MgH_2_ phases in this case.

The formed AgMg phase does not react with hydrogen, and therefore, the process of hydrogen absorption is halted. This is confirmed by hydrogen sorption kinetics studies of Alloy 8, Alloy 9, and Alloy 10 (AgMg, Ag60Mg40, and Ag_3_Mg) samples, which do not absorb hydrogen, and structural analysis performed after hydrogen sorption measurements. Similar observations have also been reported previously [14,15,19].

### 3.3. SEM and XRD Studies of Ag-Mg Alloys after Hydrogenation

**Figure 7:**
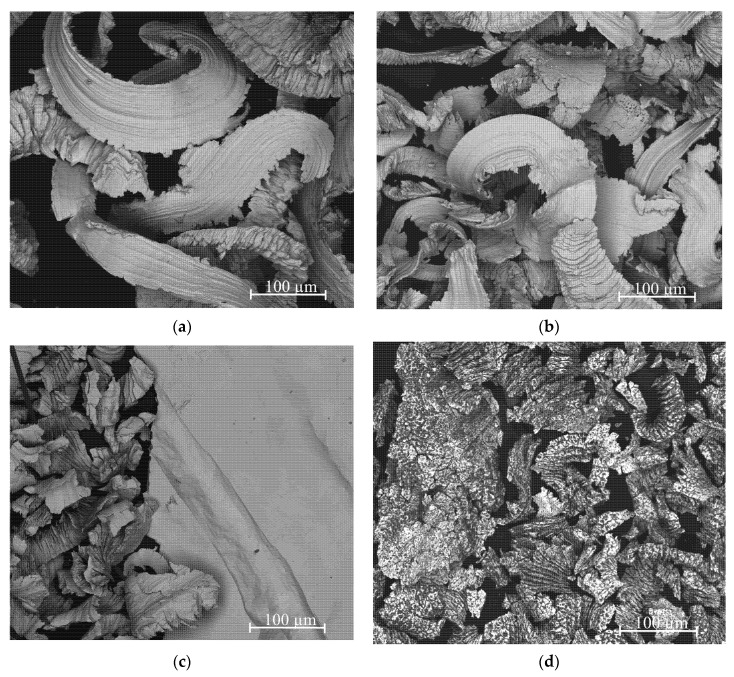

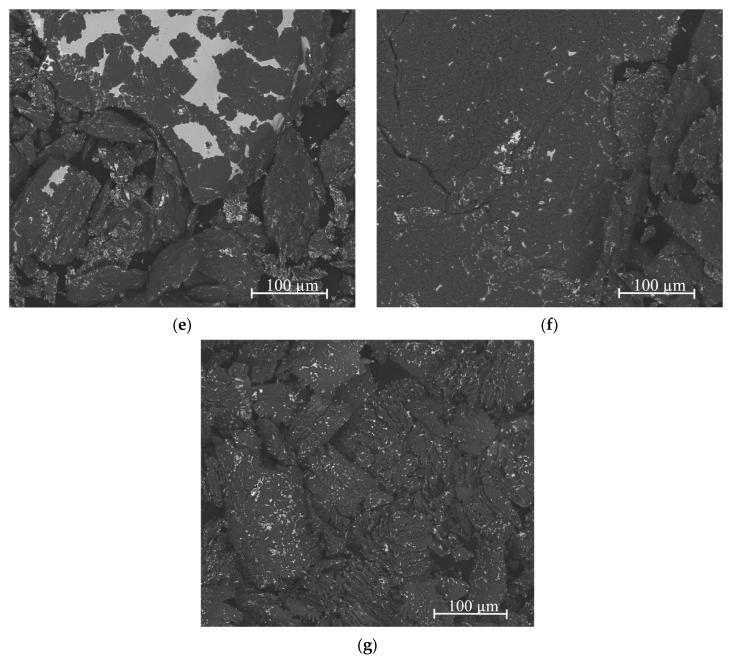
SEM microstructures of Ag-Mg alloys after hydrogenation: (**a**) Alloy 10, (**b**) Alloy 9, (**c**) Alloy 8, (**d**) Alloy 7, (**e**) Alloy 6, (**f**) Alloy 5, and (**g**) Alloy 2.

**Figure 8:**
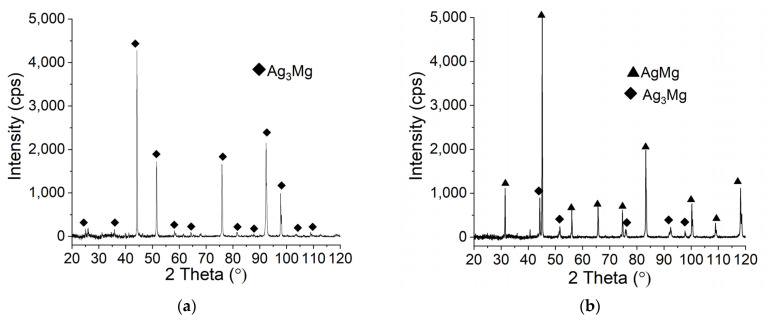

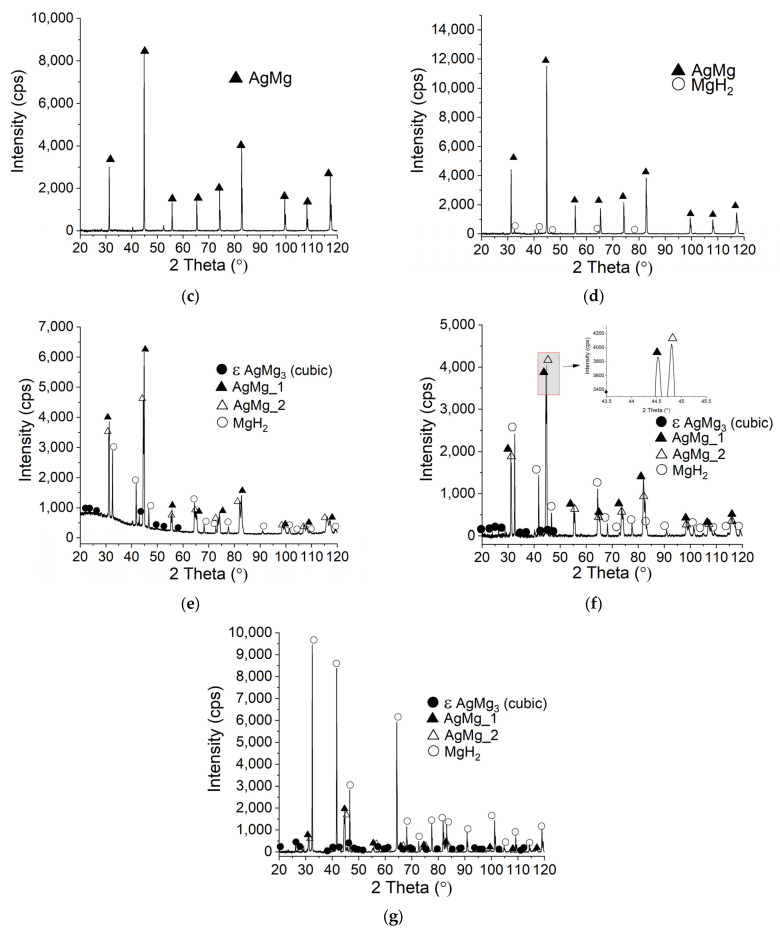
X-ray diffraction patterns for Ag-Mg samples after the hydrogenation process: (**a**) Alloy 10, (**b**) Alloy 9, (**c**) Alloy 8, (**d**) Alloy 7, (**e**) Alloy 6, (**f**) Alloy 5, and (**g**) Alloy 2.

All Ag-Mg samples after the hydrogenation process were observed using scanning electron microscopy, and X-ray phase analysis was performed in each case. Figure 7 shows the data obtained for Ag-Mg alloy samples with different Ag concentrations, starting from the highest concentration. One can observe that the first three samples with the highest Ag concentration did not show changes at the surface in contrast to the next four samples with Ag concentrations below 50 at. %, which shows surface changes, and it is very obvious that the second phase nucleates in the form of gray inclusions. The last two samples with the highest Mg contents show almost complete coverage of the surface with the dark phase. Since for BSE, darker means lighter, the formation of magnesium hydride is likely observed, which does not lead to a large contrast in the magnesium-rich samples but can be definitely observed in silver-rich samples. An important observation is that the filings maintain their form and appearance and do not break into smaller particles during this first hydrogenation. This observation is confirmed by the XRD patterns shown in Figure 8, where the first three patterns for samples with the highest silver concentration show only the existence of Ag_3_Mg and AgMg intermetallic phases and the last four patterns with Mg concentrations higher than 50 at. % show the existence of magnesium hydride (MgH_2_).

Structural studies show that the samples containing 5 wt. %, 10 wt. %, 15 wt. %, and 20 wt. % Ag (10AgMg sample is shown as a representative for this group of alloys), Alloy 5, Alloy 6, and Alloy 7, react with hydrogen to form magnesium hydride and AgMg phase. For the case of the Alloy 2 sample (Figure 8g), magnesium probably first reacts with hydrogen to form magnesium hydride, and then the AgMg_4_ phase reacts to give the ε phase, which in turn reacts to give the AgMg phase and MgH_2_. However, this observation needs to be confirmed in further studies. The visible Bragg peaks for the ε phase in the diffractograms (Figure 8e–g) can be explained by the fact that the measurement time is insufficient for the complete reaction of the ε phase transition into the AgMg phase.

**Figure 9:**
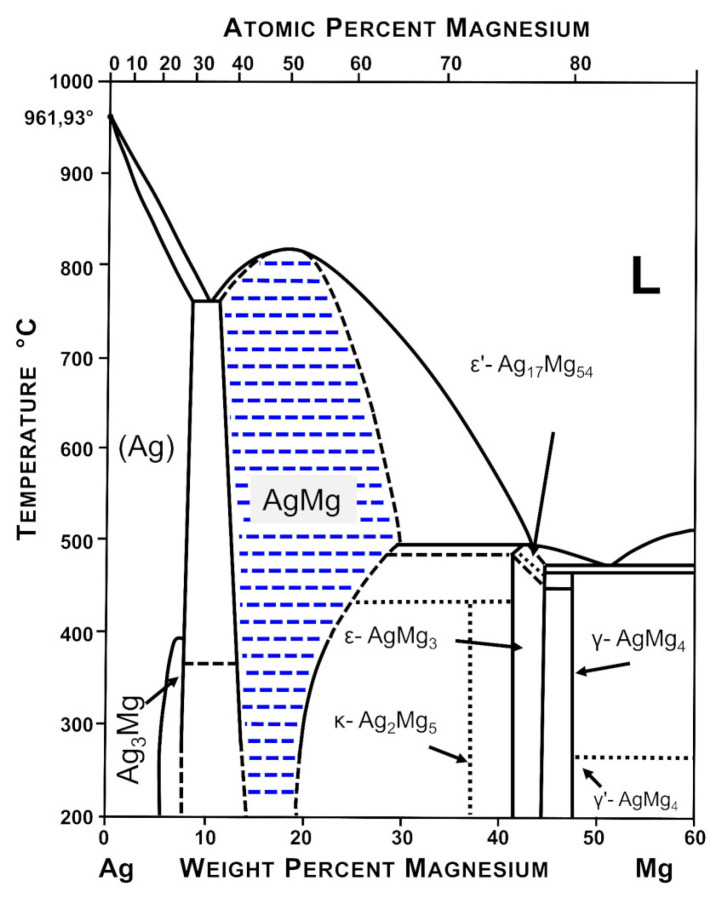
A part of the Ag-Mg phase diagram with the AgMg phase shown by the marked area [16,20,21,22].

A very interesting observation that has not been reported in the literature before is the fact that after reaction with hydrogen, AgMg is observed to exist as a mixture of two AgMg variants with slightly different lattice constants. This phenomenon is observed for samples with silver concentrations lower than 25 at. %. This behavior can be explained on the basis of the magnesium silver phase diagram that is partially shown in Figure 9. The intermetallic phase with 50 at. % Ag, namely, AgMg, exists in a relatively broad range of compositions, not a fixed stoichiometry. For that reason, a difference in the chemical composition will result in different lattice constants.

This suggests that the reaction of hydrogen with the formed AgMg may not be fully completed in the whole volume of the material. From a practical point of view, this observation is not very important however, it is relatively interesting from a cognitive point of view, as it may suggest the selective reaction of AgMg (of the proper composition) with hydrogen, while AgMg is, in general, considered not to be reactive towards hydrogen.

### 3.4. DSC and TG Measurements for Ag-Mg Alloys after Hydrogen Absorption

**Figure 10:**
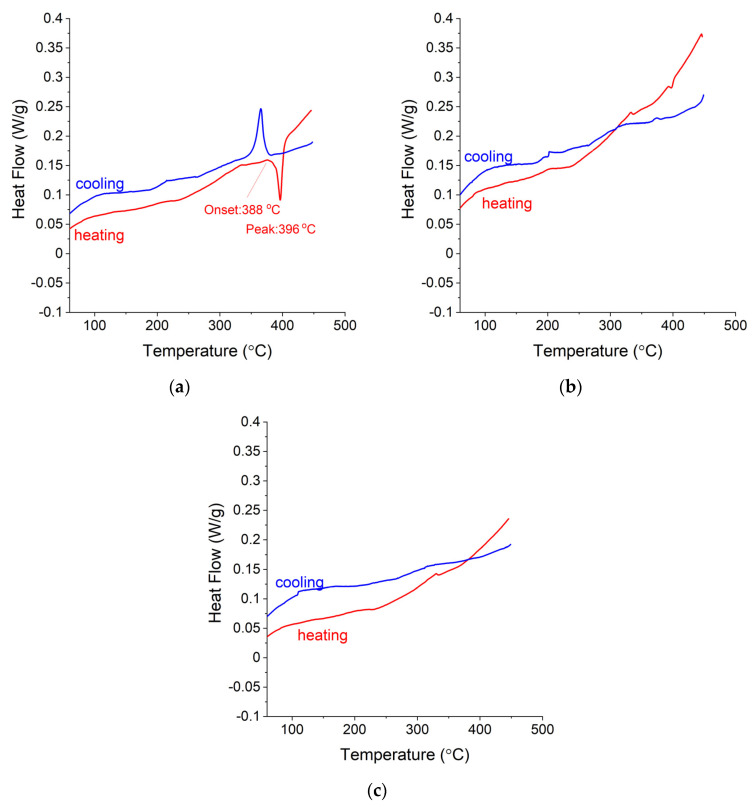
Differential scanning calorimetry profiles for (**a**) Alloy 10, (**b**) Alloy 9, and (**c**) Alloy 8.

The conclusion presented above about the reactivity of chosen alloys with hydrogen is also supported by the differential scanning calorimetry measured for the alloys after hydrogenation. The DSC plots for Alloy 1 (Ag_3_Mg) shown in Figure 10a show only one transformation peak at 388 °C (cooling) and 396 °C (heating), which is related to the phase transition of this phase, and their average temperature is the same as that reported in the literature, which correlates well with the diagram shown in Figure 1**.** No mass loss was observed at this temperature, so no hydride in any form is expected to be present. A similar lack of mass loss was observed for the two samples with Ag concentrations of up to 50 at. % Ag (Alloy 9 and Alloy 8).

**Figure 11:**
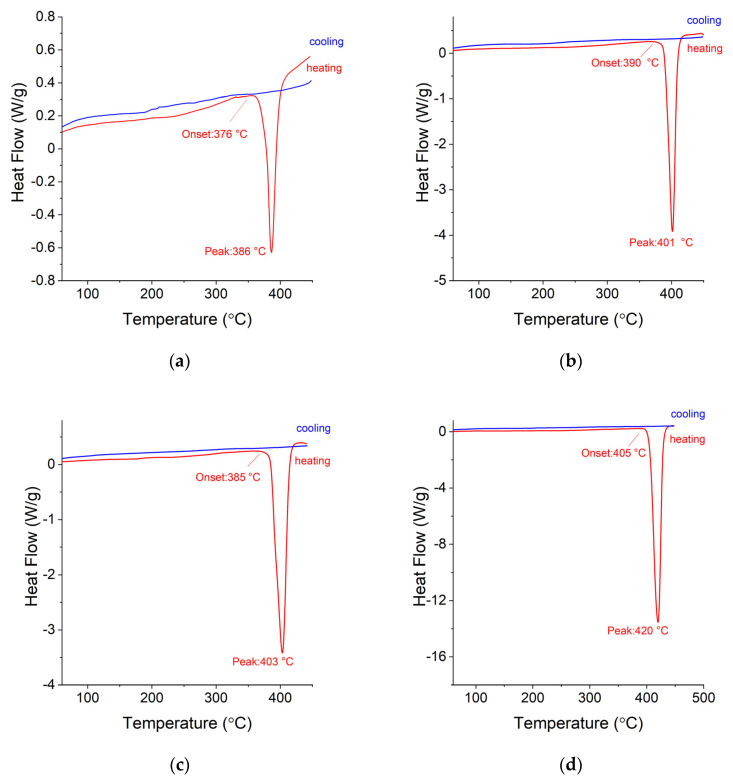
DSC curves obtained for (**a**) Alloy 7, (**b**) Alloy 6, (**c**) Alloy 5, and (**d**) Alloy 2.

**Figure 12:**
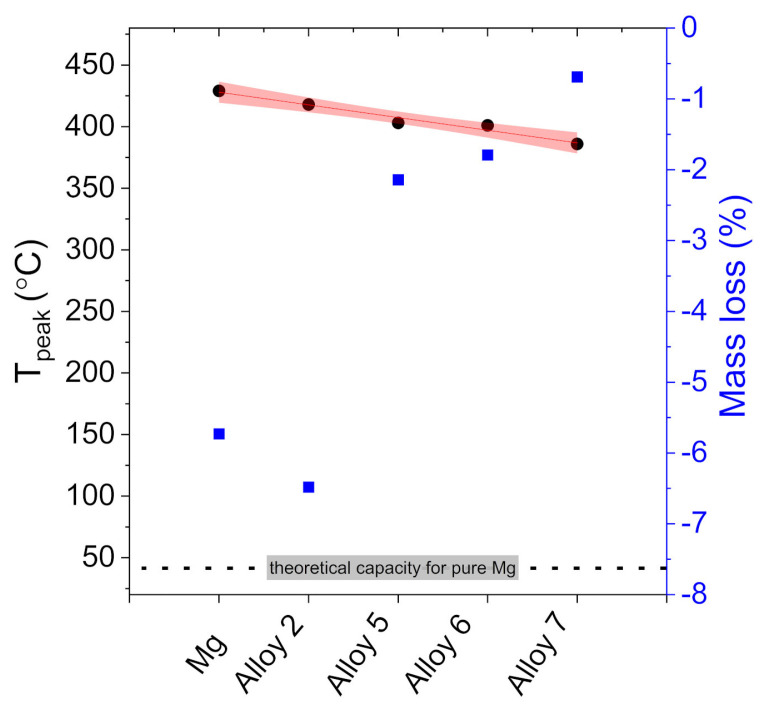
Average mass loss and decomposition peak temperatures for selected samples.

In contrast to the DSC plots shown for the three samples with the highest Ag concentration, the subsequent samples, with Mg concentrations higher than 50 at. %, show in addition, a transformation peak related to the decomposition temperature of the MgH_2_ compound. Alloy 7 shows the lowest decomposition onset temperature, which equals 376 °C (Figure 11a), and the lowest H_2_ quantity desorbed compared to the other samples (Figure 12). As the Mg concentration in the Ag-Mg samples increases, the amount of hydrogen desorbed increases, reaching 6.48 wt. %. The mass percent of desorbed hydrogen for this sample is approximately 0.5% higher than that for pure Mg. This is obviously not coherent with the theoretical capacity and is very likely only the result of the incomplete hydrogenation of pure magnesium.

A comparison of the mass loss registered during the sample decomposition and the decomposition temperature (peak temperatures) is presented in Figure 12. It can be observed that there exists a weak but noticeable correlation between the silver content and decomposition temperature. The correlation of mass loss (hydrogen content) and magnesium content is basically proportional to the amount of magnesium that can react with hydrogen (considering that AgMg is also formed).

The obtained results (temperatures) are in very good correlation with the data obtained by mechanical alloying followed by annealing [16] however, split peaks for AgMg were not observed for the case of mechanically alloyed samples, so it is probable that this specific behavior is specific only for cast material, perhaps due to the relatively large size of the filings and slow hydrogen diffusion in the material.

## 4. Conclusions

A study of the effect of Ag in Ag-Mg alloys prepared by metallurgical methods on hydrogen absorption/desorption was conducted. The final conclusions can be summarized as follows:

(1) The filings produced for the sorption study were found to show different sizes depending on whether the alloy is from a single- or two-phase region, likely due to the different mechanical properties of the alloys. The filings produced from alloys from the two-phase region are wide and thin, while those from the one-phase region are much smaller.
(2) A study of hydrogen absorption shows that alloys from the Ag-Mg system with concentrations of 50% at. Mg absorb hydrogen, and the amount of hydrogen absorbed depends on the Mg concentration in the alloy.
(3) The higher the Mg concentration, the higher the amount of absorbed hydrogen.
(4) During the reaction of alloys with hydrogen, the AgMg phase is formed, which remains in the product.
(5) Two AgMg phases are observed with different lattice parameters (observed as a peak splitting).
(6) The addition of Ag to Mg decreases the total absorption of hydrogen but slightly improves the decomposition temperature. However, the change in decomposition temperature is rather insignificant from the viewpoint of applications, especially considering the best currently known catalysts.

Although it is difficult to imagine that silver can be of any interest as a potential additive to magnesium in hydrogen storage applications, the cast alloys were found to possess properties very similar to mechanically alloyed materials, and for this reason, they may be considered a cheaper alternative.

## Figures and Tables

**Table 1:** Specification of the applied materials.

Chemical Name	Source	Purity [wt. %]	Purification Method	Analysis Method
Silver	Innovator Sp. z o. o	99.9	None	Certified purity
Magnesium	Sigma Aldrich	99.99	None	Certified purity
Argon	Air Products	99.9999	None	Certified purity
Helium	Air Products	BIP (<10 ppb O_2_)	built-in filter in the bottle	Certified purity

## Data Availability

Raw data is available upon request.

## References

[1] Pistidda C. (2020). Metals in Hydrogen Technology. Metals.

[2] Milanese C., Jensen T., Hauback B., Pistidda C., Dornheim M., Yang H., Lombardo L., Zuettel A., Filinchuk Y., Ngene P. (2019). Complex hydrides for energy storage. Int. J. Hydrogen Energy.

[3] Dematteis E.M., Berti N., Cuevas F., Latroche M., Baricco M. (2021). Substitutional effects in TiFe for hydrogen storage: A comprehensive review. Mater. Adv..

[4] Abe J.O., Popoola A.P.I., Ajenifuja E., Popoola O.M. (2019). Hydrogen energy, economy and storage: Review and recommendation. Int. J. Hydrogen Energy.

[5] Rivard E., Trudeau M., Zaghib K. (2019). Hydrogen Storage for Mobility: A Review. Materials.

[6] Ouyang L., Liu F., Wang H., Liu J., Yang X.-S., Sun L., Zhu M. (2020). Magnesium-based hydrogen storage compounds: A review. J. Alloys Compd..

[7] Baran A., Polański M. (2020). Magnesium-Based Materials for Hydrogen Storage—A Scope Review. Materials.

[8] Crivello J.C., Dam B., Denys R.V., Dornheim M., Grant D.M., Huot J., Jensen T.R., De Jongh P., Latroche M., Milanese C. (2016). Review of magnesium hydride-based materials: Development and optimisation. Appl. Phys. A.

[9] Webb C.J. (2015). A review of catalyst-enhanced magnesium hydride as a hydrogen storage material. J. Phys. Chem. Solids.

[10] Zhou C., Fang Z.Z., Ren C., Li J., Lu J. (2013). Effect of Ti intermetallic catalysts on hydrogen storage properties of magnesium hydride. J. Phys. Chem. C.

[11] Wronski Z.S., Carpenter G.J.C., Czujko T., Varin R.A. (2011). A new nanonickel catalyst for hydrogen storage in solid-state magnesium hydrides. Int. J. Hydrogen Energy.

[12] Bazzanella N., Checchetto R., Miotello A. (2011). Atoms and Nanoparticles of Transition Metals as Catalysts for Hydrogen Desorption from Magnesium Hydride. J. Nanomater..

[13] Huot J., Cuevas F., Deledda S., Edalati K., Filinchuk Y., Grosdidier T., Hauback B.C., Heere M., Jensen T.R., Latroche M. (2019). Mechanochemistry of metal hydrides: Recent advances. Materials.

[14] Si T.Z., Zhang J.B., Liu D.M., Zhang Q.A. (2013). A new reversible Mg_3_Ag–H_2_ system for hydrogen storage. J. Alloys Compd..

[15] Urretavizcaya G., Sarmiento Chávez A.C., Castro F.J. (2014). Hydrogen absorption and desorption in the Mg–Ag system. J. Alloys Compd..

[16] Pęska M., Smektalska K., Dworecka-Wójcik J., Terlicka S., Gąsior W., Gierlotka W., Dębski A., Polański M. (2021). Hydrogen sorption behavior of mechanically synthesized Mg–Ag alloys. Int. J. Hydrogen Energy.

[17] Arakcheeva A.W., Karpinskyi O.G., Kolesnichenko W.E. (1988). Crystal structure of Ag_17_Mg_54_. Crystallography.

[18] Ouyang L.Z., Cao Z.J., Yao L., Wang H., Liu J.W., Zhu M. (2014). Comparative investigation on the hydrogenation/dehydrogenation characteristics and hydrogen storage properties of Mg3Ag and Mg3Y. Int. J. Hydrogen Energy.

[19] Si T., Cao Y., Zhang Q., Sun D., Ouyang L., Zhu M. (2015). Enhanced hydrogen storage properties of a Mg-Ag alloy with solid dissolution of indium: A comparative study. Ournal Mater. Chem. A.

[20] Dębski A., Gierlotka W., Gąsior W. (2022). Calorimetric studies and thermodynamic calculations of the Ag-Mg system. J. Alloys Compd..

[21] Kudla C. (2007). Strukturell Komplexe Intermetallische. Phasen Untersuchungen an Binären und Ternären Phasen der Systeme Ag–Mg und Ag–Ga–Mg. Ph.D. Thesis.

[22] Nayeb-Hashemi A.A., Clark J.B. (1984). The Ag-Mg (Silver-Magnesium) System. Bull. Alloy. Phase Diagr..

